# The terrestrial Isopoda (Crustacea, Oniscidea) of Rapa Nui (Easter Island), with descriptions of two new species

**DOI:** 10.3897/zookeys.515.9477

**Published:** 2015-07-30

**Authors:** Stefano Taiti, J. Judson Wynne

**Affiliations:** 1 Istituto per lo Studio degli Ecosistemi, Consiglio Nazionale delle Ricerche, Via Madonna del Piano 10, 50019 Sesto Fiorentino (Florence), Italy; 2 Department of Biological Sciences, Colorado Plateau Biodiversity Center, Northern Arizona University, Box 5640, Flagstaff, Arizona 86011-5614, USA

**Keywords:** Crustacea, Isopoda, Oniscidea, new species, Rapa Nui, Easter Island, disturbance relicts, caves

## Abstract

Nine species of terrestrial isopods are reported for the Polynesian island of Rapa Nui (Easter Island) based upon museum materials and recent collections from field sampling. Most of these animals are non-native species, but two are new to science: *Styloniscus manuvaka* **sp. n.** and *Hawaiioscia rapui* **sp. n.** Of these, the former is believed to be a Polynesian endemic as it has been recorded from Rapa Iti, Austral Islands, while the latter is identified as a Rapa Nui island endemic. Both of these new species are considered ‘disturbance relicts’ and appear restricted to the cave environment on Rapa Nui. A short key to all the oniscidean species presently recorded from Rapa Nui is provided. We also offered conservation and management recommendations for the two new isopod species.

## Introduction

Rapa Nui (Easter Island) is one of the most ecologically degraded islands in Polynesia. A number of factors including geographic isolation, island size and low topographic relief (Rolett and Diamond 2004) predisposed Rapa Nui to dramatic human-induced environmental change. Between Polynesian colonization (800–1200 CE; Hunt and Lipo 2006, Shepardson et al. 2008) and prior to European contact in 1722 (McCall 1990), a catastrophic ecological shift occurred where the palm-dominated shrubland shifted to grassland (Wynne et al. 2014). By the mid-nineteenth century, most of the island was converted into a century-long sheep-grazing operation (Fischer 2005).

Contemporarily, few native plant species remain and all terrestrial vertebrates have gone extinct (Wynne et al. 2014). Researchers have described the arthropod communities of Rapa Nui as being equally impoverished (Kuschel 1963, Campos and Peña 1973, Desender and Baert 1997). Of the nearly 400 known arthropod species, only 30 species (~5%) have been identified as either endemic or indigenous with the remaining species either intentionally or accidentally introduced to the island (Wynne et al. 2014, Bernard et al. 2015).

Through fieldwork led by the second author, at least eight island endemic and two Polynesian endemic arthropod species have been recently identified (Wynne et al. 2014). These include one psocopteran (Mockford and Wynne 2013), six species of collembolans (including five new species and one Polynesian endemic; Bernard et al. 2015), one recently described collembolan later identified as endemic (Jordana and Baquero 2008; Wynne et al. 2014), and the two new species of terrestrial isopods described in this paper. All of these animals are presumed to be cave-restricted and represent disturbance relicts – organisms now restricted to a fraction of their former range due to extensive anthropogenic disturbance (Wynne et al. 2014). Given that one-third of the island’s endemic arthropod fauna appear restricted to the cave environment, this offers a unique opportunity for conservation and management.

With the exception of the Hawaiian islands (Taiti and Ferrara 1991, Taiti and Howarth 1996, 1997, Taiti 1999, Rivera et al. 2002, Taiti et al. 2003, Santamaria et al. 2013), terrestrial isopods from Polynesia are poorly known (see Jackson 1941 for a review). For Rapa Nui, only three species of terrestrial isopods were previously recorded (Fuentes 1914): *Ligia exotica* Roux, 1828, *Porcellio scaber* Latreille, 1804, and *Armadillidium vulgare* (Latreille, 1804). All of these isopods are non-native species. The purpose of this paper is to identify the terrestrial isopod fauna of Rapa Nui, including the descriptions of two new species.

## Material and methods

### Study area

Fieldwork was conducted on the Roiho lava flow, ~5 km north of the village of Hanga Roa during three research trips in 2008, 2009 and 2011. The study area is characterized by gently rolling hills (i.e., extinct scoria cones) with coastal cliff faces flanking the western-most boundary. Vegetation was grassland and invasive guava (*Psidium guajava*) shrub. Within the collapse pit and skylight entrances of most caves, several non-native tree species occurred, including fig (*Ficus* sp.), avocado (*Persea americana*), apple banana (Musa × paradisiaca), roseapple (*Syzygium jambos*), guava (*Psidium guajava*) and *Eucalyptus* spp.

### The cave environment

Caves are zonal environments often consisting of four principle zones: (1) an entrance (or light) zone representing a combination of both surface and cave climatic conditions; (2) a twilight zone where light is diminished and surface climate conditions are progressively dampened; (3) a transition zone characterized by complete darkness with a further diminished influence of surface climate conditions; and, (4) a deep zone (usually the deepest portion of the cave) where environmental conditions (e.g., complete darkness, temperature, and air flow) remain relatively stable over time and the evaporation rate is negligible (Howarth 1980, 1982). For each isopod detected within caves, we provide a zone designation in the “type material examined” section.

### Sampling

Cave and surface sampling was conducted. Research teams (led by the second author) systematically sampled 10 caves during three research trips (16–21 August 2008; 28 June–17 July 2009; and 01–07 August 2011). Four methodologies (pitfall traps, time-constrained searches, opportunistic collecting, and timed direct intuitive searches) were applied to sample 10 caves during the first two trips. Pitfall trap construction consisted of two 946-ml stacked plastic containers (13.5 cm high, 10.8-cm-diameter rim and 8.9-cm base). A teaspoon of peanut butter placed in the bottom of the exterior container was used as bait. The bottom of the interior container had several dozen holes to allow the bait to “breathe” to attract arthropods. Traps were deployed for three to four days.

Time-constrained searches involved estimating a one-meter radius around each pitfall trap sampling station and then conducting a timed search. Searches were conducted for one to three minutes (one minute if no arthropods were observed, three if arthropods were detected) before pitfall trap deployment and prior to trap removal.

Opportunistic collection involved collecting arthropods as encountered – while deploying and removing pitfall traps, and between timed searches. During these intervals, personnel searched the ground, walls and ceilings as they walked the length of each cave. In five caves (where all the collecting methodologies were applied), we also conducted timed direct intuitive searches (DIS) of fern-moss gardens by gently combing through the fern and moss and looking beneath rocks for 40 search-minutes per garden (two observers × 20 minutes per observer). In four additional caves, we limited sampling to DIS within fern-moss gardens only (two observers × 20 minutes per observer).

During the last research trip to the island, the deep zones of four of the caves were sampled via bait sampling and DIS. Three types of baits were placed directly on the ground and within cracks and fissures on cave walls, ceilings and floors: sweet potato (*Ipomoea batatas*), chicken and fish entrails, and small branches from local hibiscus (*Hibiscus rosa-sinensis*) and Gaoho (*Caesalpinia major*) shrubs. Two to three stations of each bait type were deployed, for four to five days, within the deep zone(s) of each cave. At proximity to bait sampling arrays, we also conducted one DIS by searching the cave floor for 10 minutes within a 1-m^2^ area.

From 28 June through 08 July 2009 (total of 10 days), we deployed two 15 x 20 meter surface pitfall trapping grids. Surface Grid 1 (with trap numbers 1 - 20) was established inland at the approximate center of our study area. Surface Grid 2 (with trap numbers 21 - 40) was deployed at the western extent of the study area (~250 m from the coastal cliff face). All pitfall traps were countersunk to ground surface with trap spacing at 5 m between each trap.

For additional information on sampling refer to Wynne et al. (2014) at: http://www.bioscience.oxfordjournals.org/lookup/suppl/doi:10.1093/biosci/biu090/-/DC1

### Cave codes

We recognize standard practice for locality information is to provide geographical coordinates to facilitate future collecting and interpretation. However, Chilean park officials have requested that neither cave names nor coordinates be included due to cultural and natural resource sensitivities of caves. In place of cave names, we used cave codes supplied by CONAF – Parque Nacional Rapa Nui. A copy of this paper, which includes a table of cave names with associated cave codes, is on file with CONAF – Parque Nacional Rapa Nui headquarters Hanga Roa, Easter Island, and CONAF, Jefe Departamento, Diversidad Biológica, Gerencia de Areas Protegidas y Medio Ambiente, Santiago, Chile.

### Preservation, mounting, observation

All material was preserved in 95% ethanol. Identifications were based on morphological characters with the use of micropreparations. Line drawings were made with the aid of a camera lucida mounted on Wild M5 and M20 microscopes. Whole-specimen images were captured using a 1.1 MP Canon 5D Mark II (with a 65 mm zoom lens) mounted on a Visionary Digital BK Lab Plus camera mounting system. We used the program Zerene Stacker to merge images into a composite image. Photoshop CS5 was used for image post-processing.

### Museum abbreviations

AMNH American Museum of Natural History, New York, USA;

BPBM Bernice P. Bishop Museum, Honolulu, Hawai‘i, USA;

MNHN Museo Nacional de Historia Natural, Santiago, Chile;

MZUF Museo di Storia Naturale, sezione di Zoologia, dell’Università di Firenze, Florence, Italy;

YPM Peabody Museum of Natural History, Yale University, New Haven, Connecticut, USA.

## Systematic account

### Family Ligiidae

#### Genus *Ligia* Fabricius, 1798

##### Ligia exotica

Taxon classificationAnimaliaIsopodaLigiidae

Roux, 1828

Ligyda exotica ; Fuentes 1914: 315.

###### Remarks.

The record of this species by Fuentes (1914) needs to be confirmed since this littoral species has often been confused with other species in the past. Unfortunately, no specimens of *Ligia* have been recently collected from Rapa Nui, most likely due to lack of investigations along the littoral zones of the island.

###### Distribution.

Pantropical.

### Family Styloniscidae

#### Genus *Styloniscus* Dana, 1853

##### Styloniscus manuvaka sp. n.

Taxon classificationAnimaliaIsopodaStyloniscidae

http://zoobank.org/00706DDB-9E9C-4DEF-AEA5-E9289287B7AB

Figs 1A , 2 , 3 , 4

Styloniscus sp.; Wynne et al. 2014: 713, 714, fig. 2b.

###### Type material examined.

Chile, Rapa Nui: 1 ♂ holotype, 2 ♂♂, 2 ♀♀, 1 juv. paratypes (MNHN), Mahunga Hiva Hiva, Cave Q15-070, fern-moss garden (entrance zone), direct intuitive search, 10.VII.2009, leg. J.J. Wynne; 2 ♂♂, 1 ♀, 2 juvs. paratypes (MZUF), same location, 50 m from entrance, direct intuitive search (on decomposing tree branches; twilight zone), 6.VIII.2011, leg. J.J. Wynne; 1 ♀ paratype (MNHN), same data; 1 ♂, 1 ♀ paratypes (BPBM), Mahunga Hiva Hiva, Cave Q15-074, skylight entrance (1^st^ entrance NE of main entrance; entrance zone), 3.VII.2009, leg. J.J. Wynne; 1 ♀ paratype (BPBM), Mahunga Hiva Hiva, Cave Q15-119, timed search at trap 4A, 5.VII.2009, leg. J.J. Wynne; 1 ♂ paratype (BPBM), same location, Zone 2 (approx. cave deep zone), trap, fish entrails 1, 6.VIII.2011, leg. J.J. Wynne; 1 ♀ paratype (BPBM), Mahunga Hiva Hiva, Cave Q15-071, Zone 2 (approx. cave deep zone), bait trap, fish entrails 1, 7.VIII.2011, leg. J.J. Wynne; 1 ♀ paratype (BPBM), Cave Q15-067, fern-moss garden (entrance zone), direct intuitive search, 4.XII.2008, leg J.J. Wynne.

###### Additional material examined.

French Polynesia, Bass Islands (Austral Islands), Rapa Iti Island: 4 ♂♂ (YPM), Pumarua-Maurua Ridge, Pumarua and some west, 500-620 m, from dead leaves of the bird’s nest fern, *Asplenium nidus*, 9.I.1980, leg. G. Paulay.

###### Description.

**Figure 1.**
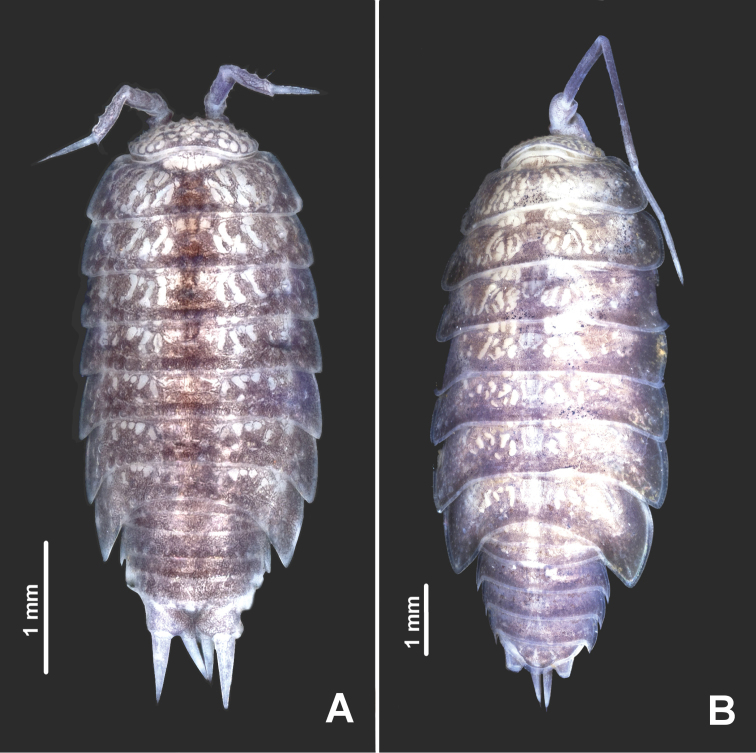
*Styloniscus manuvaka* sp. n.: **A** ♀ paratype in dorsal view. *Hawaiioscia rapui* sp. n.: **B** ♀ paratype in dorsal view.

**Figure 2.**
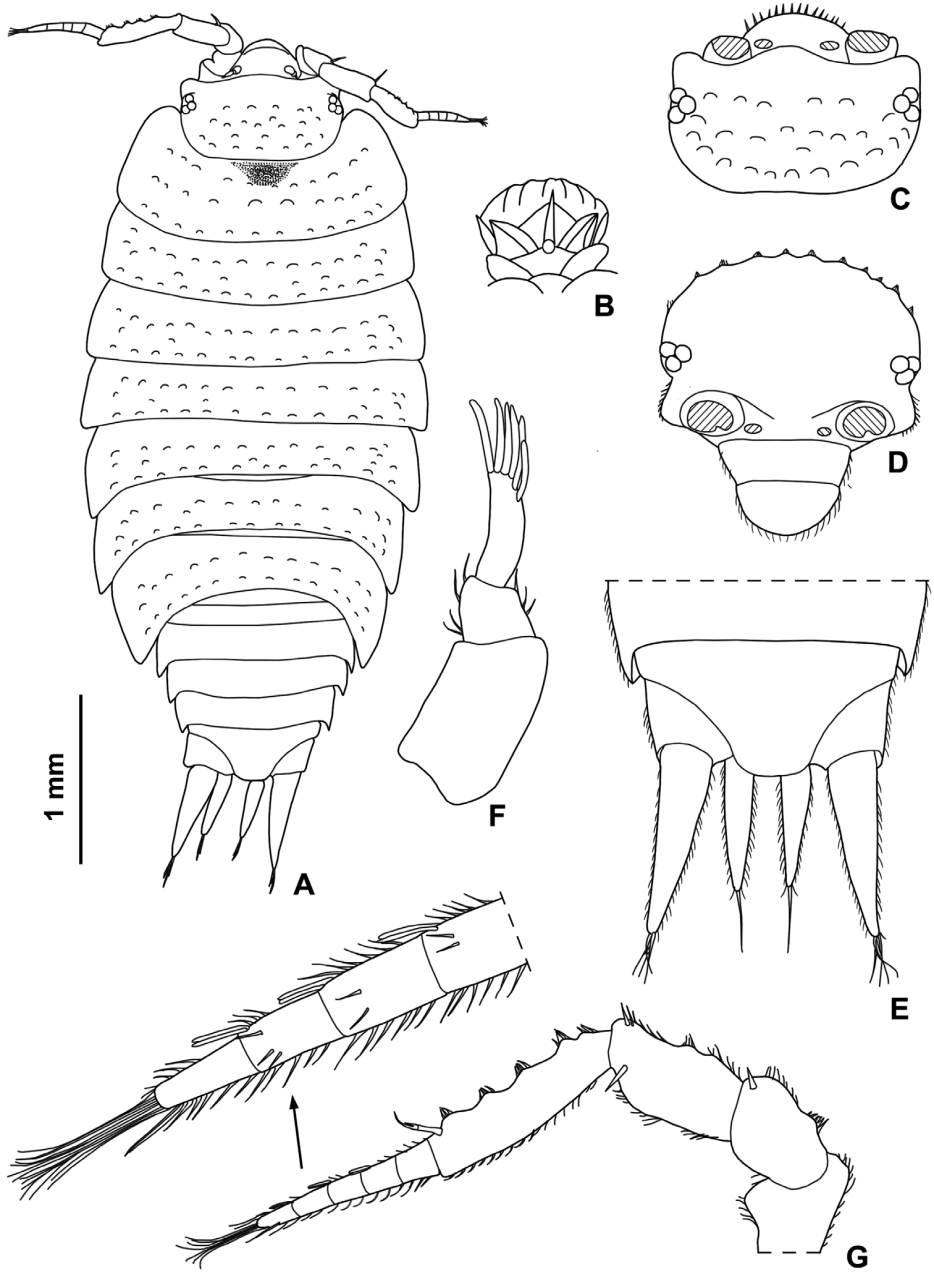
*Styloniscus manuvaka* sp. n., ♀ paratype: **A** adult specimen in dorsal view **B** dorsal scale-seta **C** cephalon in dorsal view **D** cephalon in frontal view **E** pleonite 5, telson and uropods **F** antennula **G** antenna.

**Figure 3.**
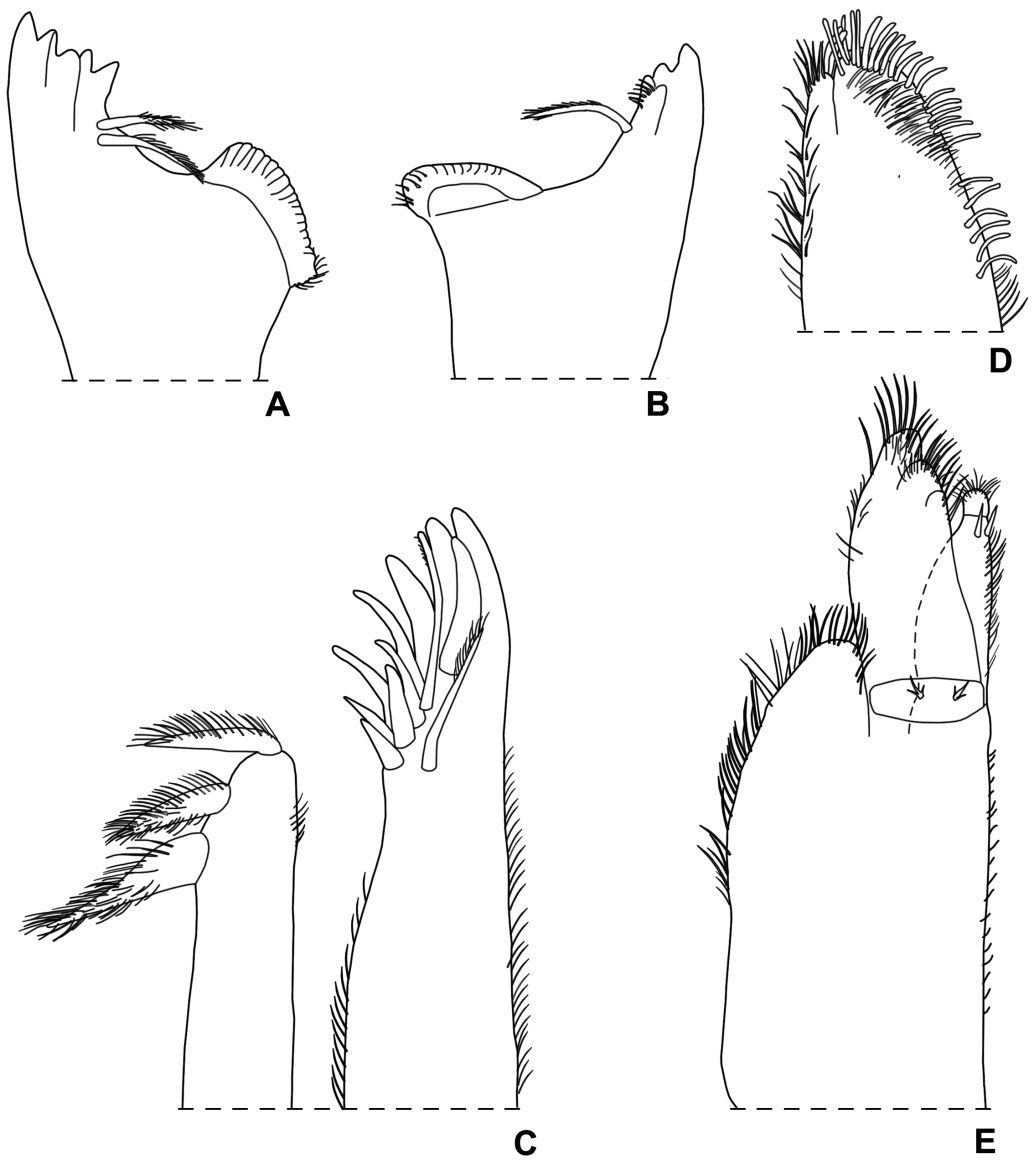
*Styloniscus manuvaka* sp. n., ♀ paratype: **A** left mandible **B** right mandible **C** maxillula **D** maxilla **E** maxilliped.

**Figure 4.**
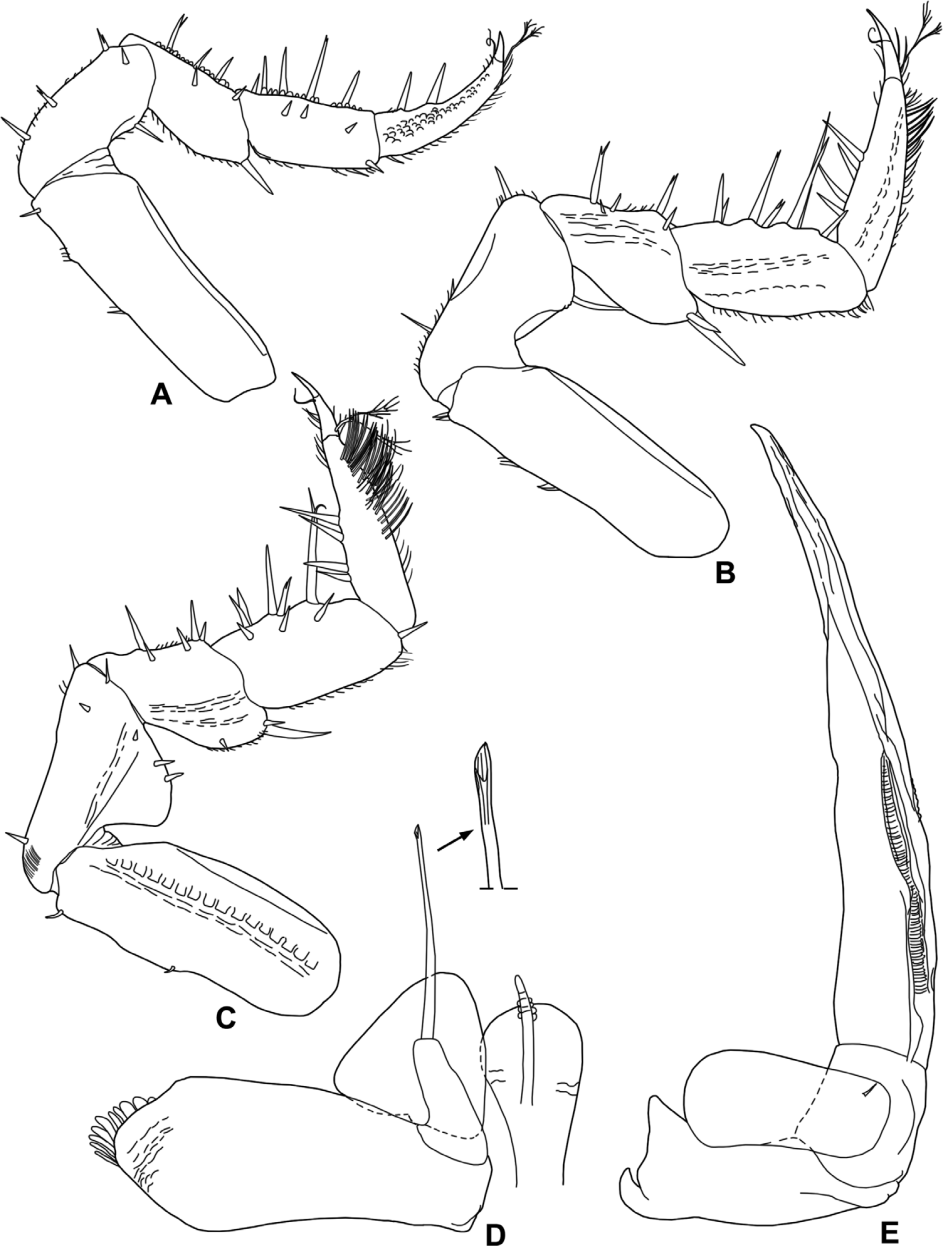
*Styloniscus manuvaka* sp. n., ♂ paratype: **A** pereopod 1 **B** pereopod 6 **C** pereopod 7 **D** genital papilla and pleopod 1 **E** pleopod 2.

Maximum length: ♂ 4 mm, ♀ 4.2 mm. Dorsum brown with the usual yellow muscle spots (Fig. 1A). Body ovoid with pleon narrower than pereon (Figs 1A, 2A). Vertex and pereon distinctly granulated with granulations arranged on three rows on pereonite 1 and two rows on pereonites 2-7; pleon and telson smooth. Dorsal surface with scale-setae as in Fig. 2B. Cephalon (Fig. 2C, D) with obtuse middle lobe slightly protruding frontwards compared with rounded lateral lobes; eye consisting of three ommatidia in a triangle. Pleonites 3-5 reduced with small posterior points. Telson (Fig. 2E) with concave sides and truncate apex. Antennula (Fig. 2F) with second article shorter than first and third; third article with 6 long aesthetascs at apex. Antenna (Fig. 2G) with flagellum as long as fifth article of peduncle; flagellum cone-shaped, consisting of 5 articles with the second, third and fourth article bearing two aesthetascs each. Left mandible (Fig. 3A) with 2 penicils; right mandible (Fig. 3B) with 1 penicil. Maxillula (Fig. 3C) outer branch with 10 simple teeth and 2 long stalks; inner branch with 3 penicils. Maxilla (Fig. 3D) apically bilobate, inner lobe wider than outer lobe and bearing strong setae on its margin. Maxilliped (Fig. 3E) endite with a stout apical penicil; basal article of the palp with 2 setae. Pereopods 6 and 7 with a distinct water conducting system (Fig. 4B,C) on merus, carpus and propodus, and on basis, ischium and merus, respectively.

Male. Pereopod 1 (Fig. 4A) merus and carpus with a line of short scales on sternal margin. Pereopod 7 (Fig. 4C) ischium enlarged in the distal part, forming a flat rounded lobe with two short and stout setae on tergal margin, sternal margin almost straight; propodus with numerous long and thin setae on tergal margin. Genital papilla (Fig. 4D) with rounded and enlarged distal part. Pleopod 1 (Fig. 4D) exopodite triangular, as wide as long, with rounded posterior margin; endopodite with flagelliform distal segment, about twice as long as basal one and slightly enlarged at apex. Pleopod 2 (Fig. 4E) exopodite very short, rectangular, about twice wider than long; endopodite with distal segment about seven times longer than basal one, with tapering apical part slightly bent outwards, acute apex.

###### Etymology.

The species name is a combination of two Rapanui terms, *manu* and *vaka*. *Manu* is “bug” and *vaka* is “canoe” or “boat”; when combined this translates to “canoe bug.” Based upon the identification of this species, and a collembolan (*Lepidocyrtus olena* Christiansen & Bellinger, 1992) previously known from the Hawaiian Islands only, Wynne et al. (2014) suggested both of these animals may have been dispersed by the ancient Polynesians as they transported and transplanted cultivars (called “canoe plants”), such as banana, taro and sugar cane, throughout the South Pacific islands.

###### Remarks.

At present the genus *Styloniscus* includes about 45 species distributed in the tropics and the southern hemisphere (Schmalfuss 2003; Nunomura 2007; Taiti 2014). The new species is characterized by the male pereopod 7 ischium enlarged in the distal part with a flat rounded lobe. A similar character is present also in a species from Omaio, North Island, New Zealand, identified by Vandel (1952) as *Styloniscus otakensis* (Chilton, 1901). The specimens redescribed and illustrated by Vandel certainly do not belong to *Styloniscus otakensis* according to the redescription of this species provided by Green (1971) on the basis of the type material studied by Chilton (1901) and on topotypic material (Dunedin, South Island). In fact, the male pereopod 7 ischium does not show any distinct lobe (compare fig. 31 in Green 1971 with fig. 37 in Vandel 1952), and the shapes of the male pleopod 1 exopodite and pleopod 2 endopodite are significantly different (compare figs 29 and 30 in Green 1971 with figs 38 and 39A in Vandel 1952). Thus, the specimens from Omaio must belong to a distinct species yet to be named. *Styloniscus manuvaka* sp. n. differs from *Styloniscus otakensis* sensu Vandel nec Chilton in having 6 instead of 5 aesthetascs at the apex of the antennula, 5 instead of 4 flagellar articles of the antenna, the male pereopod 7 ischium with two, instead of one, stout setae on the tergal margin, and the male pleopod 2 endopodite with a thicker distal part.

On Rapa Nui, *Styloniscus manuvaka* sp. n. is presently restricted to the cave environment, but is not troglomorphic (cave-adapted). This animal was detected within the fern-moss gardens (entrance zone) of three caves, but also occurred within the twilight and cave deep zones. This species was not detected during the surface sampling work conducted in 2009, nor has it been identified during previous invertebrate inventory work (e.g., Fuentes 1914, Olalquiaga 1946, Kuschel 1963, Campos and Peña 1973). The species also occurs on Rapa Iti, Bass Islands, where it is not restricted to the cave environment. This species is considered a Polynesian endemic and it might be present also on other Pacific islands.

###### Distribution.

Presently known from Rapa Nui and Rapa Iti.

### Family Philosciidae

#### Genus *Hawaiioscia* Schultz, 1973

##### Hawaiioscia rapui sp. n.

Taxon classificationAnimaliaIsopodaPhilosciidae

http://zoobank.org/56E14D72-3CF5-4E39-A655-15659F01B67A

Figs 1B , 5 , 6 , 7

Hawaiioscia sp.; Wynne et al. 2014: 714, 716, fig. 2a.

###### Type material examined.

Chile, Rapa Nui: 1 ♂ holotype, 2 ♀♀ paratypes (MNHN), Mahunga Hiva Hiva, Cave Q15-034, pitfall trap 5A (twilight zone) 12.VII.2009, leg. J.J. Wynne; 1 ♀ paratype (MZUF), 1 ♀ paratype (BPBM), same data, pitfall trap 7A (approx. deep zone); 1 ♂ Paratype (MZUF), Mahunga Hiva Hiva, Cave Q15-076/078, pitfall trap 2C (light zone), 4.VII.2009, leg. J.J. Wynne.

###### Description.

**Figure 5.**
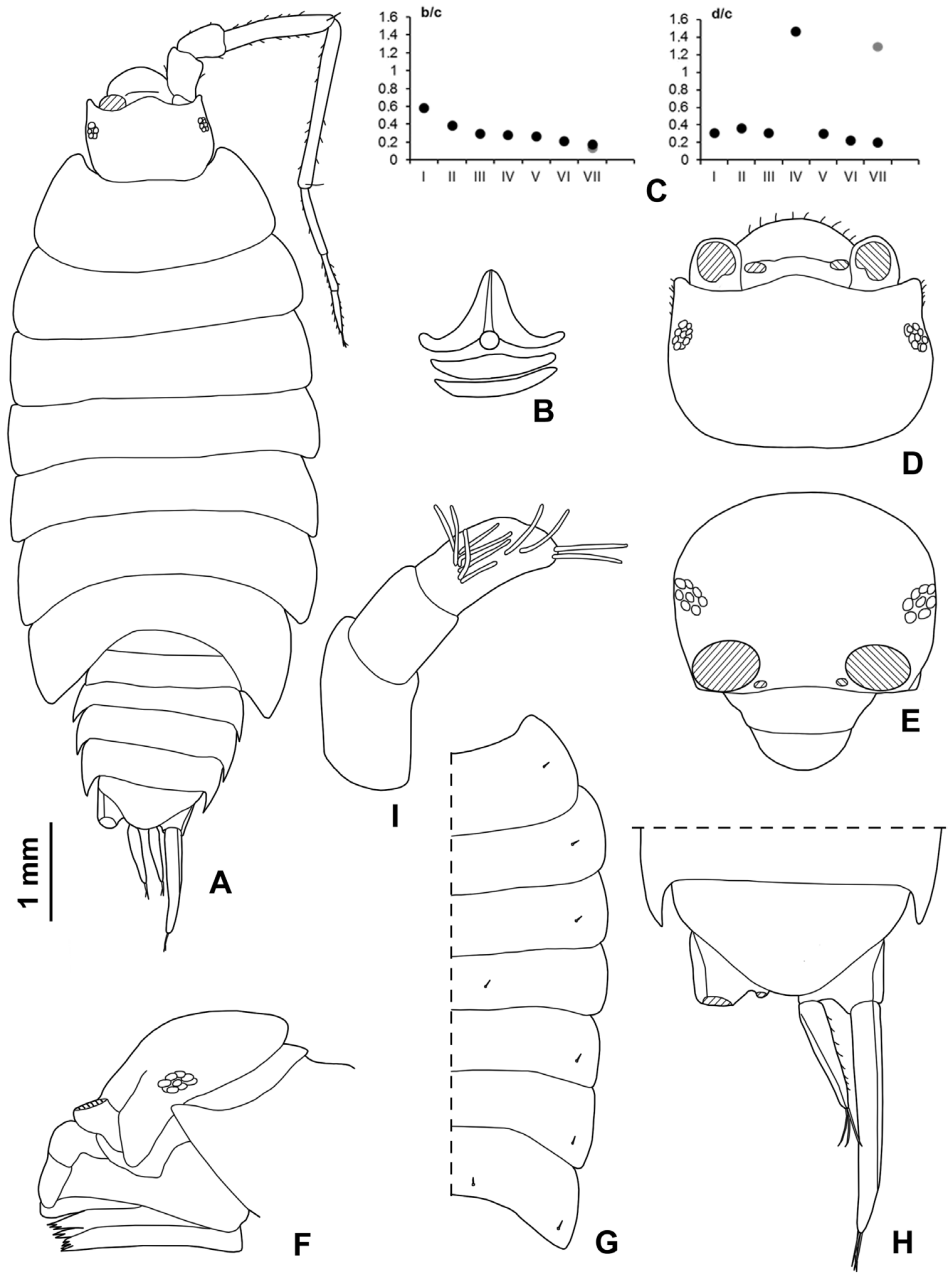
*Hawaiioscia rapui* sp. n., ♂ holotype: **A** adult specimen in dorsal view. ♀ paratype: **B** dorsal scale-seta **C** co-ordinates of noduli laterales **D** cephalon in dorsal view **E** cephalon in frontal view **F** cephalon in lateral view **G** pereonites with noduli laterales **H** pleonite 5, telson and uropods **I** antennula.

**Figure 6.**
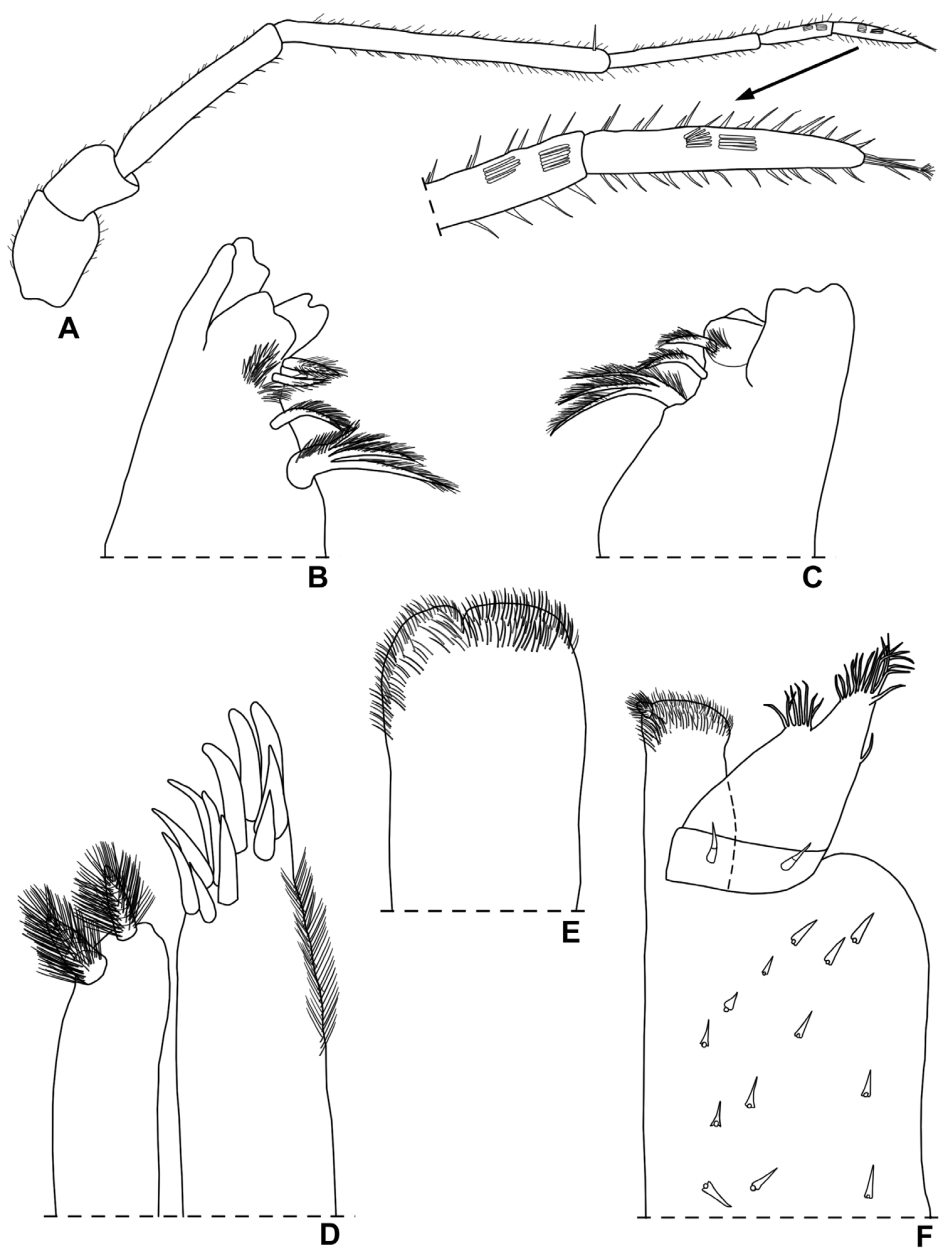
*Hawaiioscia rapui* sp. n., ♀ paratype: **A** antenna **B** left mandible **C** right mandible **D** maxillula **E** maxilla **F** maxilliped.

**Figure 7.**
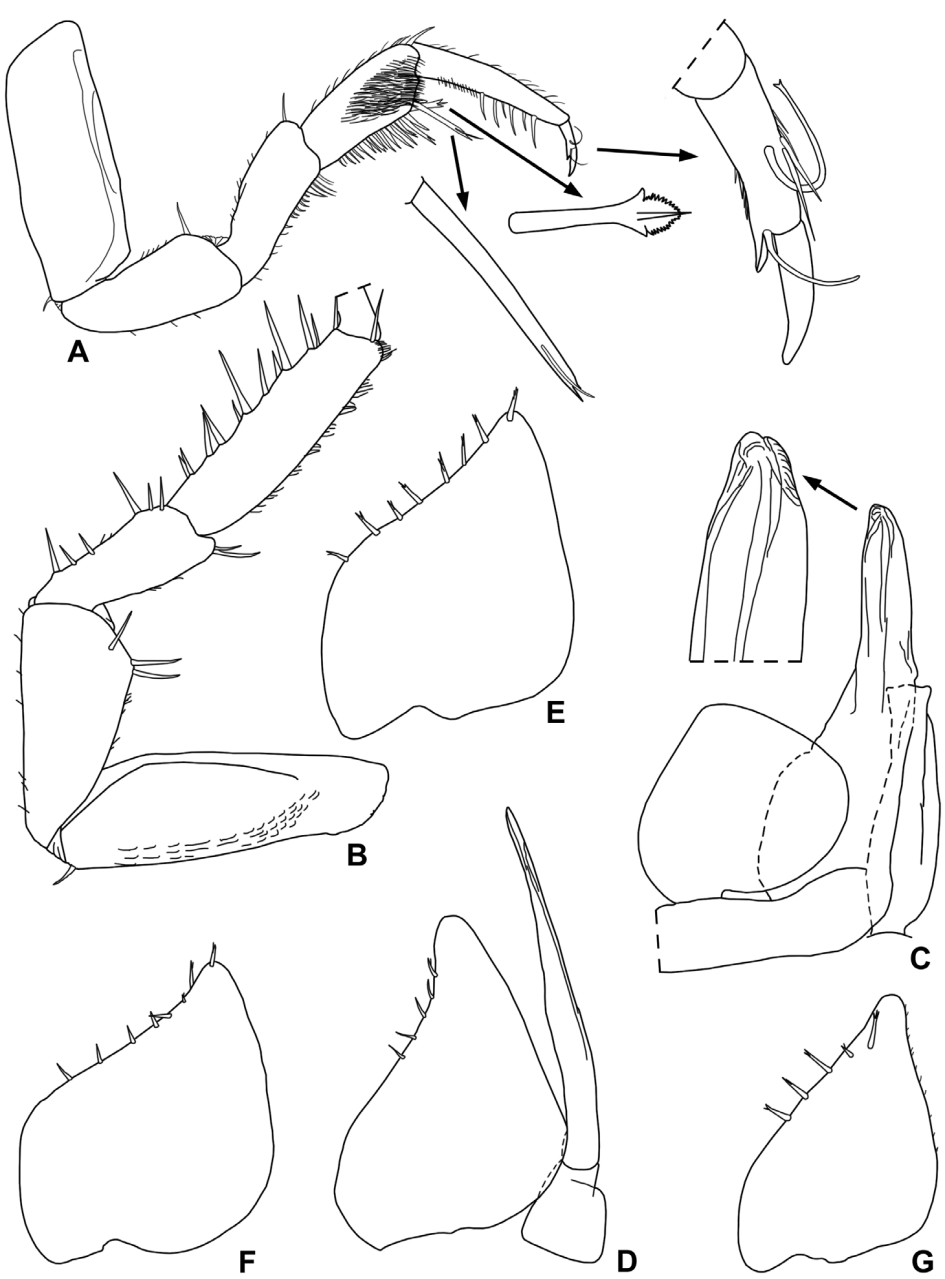
*Hawaiioscia rapui* sp. n., ♂ paratype: **A** pereopod 1 **B** pereopod 7 **C** genital papilla and pleopod 1 **D** pleopod 2 **E** pleopod 3 exopodite **F** pleopod 4 exopodite **G** pleopod 5 exopodite.

Maximum length: ♂ and ♀ 7.5 mm. Dorsum light brown with the usual muscle spots (Fig. 1B). Body flat, ovoidal, with pleon narrower than pereon, outline as in Fig. 5A. Dorsal body surface finely granulated with small triangular scale-setae (Fig. 5B). Pereonites with no sulcus marginalis, gland pores absent. Noduli laterales (Fig. 5C, G) clearly visible, inserted on a small tubercle and disposed as follows: two on the cephalic vertex, one per side on pereonites 1-6 with that on the fourth pereonite much more distant from the lateral margin of the segment, and two per side on pereonite 7. Cephalon (Fig. 5D–F) with short triangular lateral lobes not protruding frontwards compared with the obtuse middle lobe; frontal and supra-antennal lines absent; eyes small, consisting of eight ommatidia. Pleon epimera reduced but with distinct posterior points (Fig. 5A,H). Telson (Fig. 5H) triangular, about twice as wide as long, with broadly rounded apex. Antennula (Fig. 5I) of 3 articles, second article slightly shorter than first and third; third article bearing two rows of 7 and 2 aesthetascs each, and 2 apical aesthetascs. Antenna (Fig. 6A) long and thin, reaching back rear margin of pereonite 6; flagellum as long as fifth peduncular article, first flagellar article distinctly longer than second and third, with two rows of 4 to 6 aesthetascs on each second and third article. Mandibles (Fig. 6B,C) with molar penicil semidichotomized, i.e. consisting of 3-4 setae on a common stem; left mandible with 2+1 and right mandible with 1+1 free penicils. Maxillula (Fig. 6D) outer branch with 5+6 teeth, all simple; inner branch with two stout subequal penicils. Maxilla (Fig. 6E) apically setose and bilobate with outer lobe wider than inner one. Maxilliped (Fig. 6F) endite apically setose and bearing a large penicil at medial corner, proximal article of palp bearing 2 strong setae. Pereopods with elongated articles and flagelliform dactylar and ungual setae (Fig. 7A). Pleopodal exopodites with no trace of respiratory structures. Uropod (Fig. 5H) protopod with a /\-shaped groove on outer margin; insertion of endopodite slightly proximal to that of exopodite.

Male. Pereopod 1 carpus with a brush of trifid spines on sternal margin (Fig. 7A). Pereopod 7 (Fig. 7B) with no peculiar modifications, ischium with sternal margin straight. Pleopod 1 (Fig. 7C) exopodite cordiform, with a broadly rounded apex; endopodite with thickset distal part, straight with rounded apex. Pleopod 2 (Fig. 7D) with exopodite triangular, shorter than endopodite and bearing 5 setae on ouer margin. Pleopods 3-5 exopodite as in Fig. 7E–G.

###### Etymology.

The new species is named after Sergio Rapu Haoa, a humanitarian who has furthered cultural and archeological knowledge of Rapa Nui. Sergio was Rapa Nui’s first governor of Rapanui descent and the first director and curator of Museo Antropológico P. Sebastián Englert on Rapa Nui. He is also a world-renowned Rapa Nui archaeologist and purveyor of Rapa Nui culture. He graciously provided logistical support to the second author and his research teams while on Rapa Nui.

###### Remarks.

Prior to discovering this new species, the genus *Hawaiioscia* consisted of four troglomorphic species restricted to lava tube caves on the Hawaiian Islands (Schultz 1973; Taiti and Howarth 1997): *Hawaiioscia parvituberculata* Schultz, 1973 from Maui, *Hawaiioscia microphthalma* Taiti & Howarth, 1997 from O‘ahu, *Hawaiioscia paeninsulae* Taiti & Howarth, 1997 from Moloka‘i, and *Hawaiioscia rotundata* Taiti & Howarth, 1997 from Kaua‘i. No epigean species in this genus were previously known. The new species shows all the characters of the genus *Hawaiioscia* with the sole exception of the molar penicil of the mandible which is semidichotomized instead of simple as in all the others species from Hawai‘i. Considering that all the most important characters (number and position of noduli laterales, maxillular teeth, penicil on maxillipedal endite, uropod and shape of male pleopod 1) are shared with all the other *Hawaiioscia* species, we include the new species in this genus.

Specimens from this new species were collected from both within the entrance zone of one cave and the twilight zone of another cave. It is important to note, this species does not have troglomorphic characteristics, such as body depigmentation or eye reduction as do other congeners within *Hawaiioscia*. However, as with *Styloniscus manuvaka* sp. n., this new species was not detected during the surface sampling effort, nor has it been previously identified by earlier entomological surveys of the island. Thus, we believe this animal to be restricted to cave environment on Rapa Nui.

###### Distribution.

Presently endemic to Rapa Nui.

### Family Platyarthridae

#### Genus *Trichorhina* Budde-Lund, 1908

##### Trichorhina tomentosa

Taxon classificationAnimaliaIsopodaPlatyarthridae

(Budde-Lund, 1893)

###### Material examined.

Chile, Rapa Nui: 1 ♀ (BPBM), Mahunga Hiva, Cave Q15-074, pitfall trap 1B (light zone), 30.VI.2009, leg. J.J. Wynne.

###### Distribution.

Pantropical. Introduced to greenhouses worldwide.

### Family Porcellionidae

#### Genus *Porcellionides* Miers, 1877

##### Porcellionides pruinosus

Taxon classificationAnimaliaIsopodaPorcellionidae

(Brandt, 1833)

###### Material examined.

Chile, Rapa Nui: 2 ♂♂, 3 ♀♀ (AMNH 18360), Cannibal Cave, 21.VIII.1999, leg. C. Boyko, J. Tanacredi, S. Reanier, H. Tonnemacher and S. Lopez; 1 ♀ (BPBM), Mahunga Hiva Hiva, Cave, Q15-074, time search at 1A (light zone), 30.VI.2009, leg. J.J. Wynne; 1 ♂, 3 ♀♀ (BPBM), same location, leaf litter beneath skylight, (3^rd^ entrance NW of main entrance; entrance zone), direct intuitive search, 2.VIII.2011, leg. J.J. Wynne; 1 ♀ (BPBM), Mahunga Hiva Hiva, Cave Q15-067, fern-moss garden (entrance zone), direct intuitive search, 10.VII.2009, leg. J.J. Wynne; 1 ♂ (BPBM), Mahunga Hiva Hiva, Cave Q15-070, fern-moss garden (entrance zone), direct intuitive search, 10.VII.2009, leg. J.J. Wynne.

###### Distribution.

Cosmopolitan species of Mediterranean origin.

#### Genus *Porcellio* Latreille, 1804

##### Porcellio scaber

Taxon classificationAnimaliaIsopodaPorcellionidae

Latreille, 1804

Porcellio scaber ; Fuentes 1914: 315; Wynne et al. 2014: 716.

###### Material examined.

Chile, Rapa Nui: 1 ♀ (AMNH 18362), VIII.1999, leg. C. Boyko, J. Tanacredi, S. Reanier, H. Tonnemacher and S. Lopez; 2 ♂♂, 1 ♀ (AMNH 18363), Maunga Tangaroa, 20.VIII.1999, leg. C. Boyko, J. Tanacredi, S. Reanier, H. Tonnemacher and S. Lopez; 2 ♂♂, 3 ♀♀ (AMNH 18365), Ana te Pahu, 21.VIII.1999, leg. C. Boyko, J. Tanacredi, S. Reanier, H. Tonnemacher and S. Lopez; 2 ♂♂, 13 ♀♀ (AMNH 18364), Poike region, 25.VIII.1999, leg. C. Boyko, J. Tanacredi, S. Reanier, H. Tonnemacher and S. Lopez; 1 ♂ (BPBM), Mahunga Hiva Hiva, surface in front of Cave Q15-038, opportunistic collection (eastern-most collapse pit, southern extent near cave entrance), 18.VIII.2008, J.J. Wynne; 1 ♂ (BPBM), Mahunga Hiva Hiva, surface grid 2, 27°06'41.3"S, 109°25'09.2"W, pitfall trap 21, 10.VII.2009, leg. J.J. Wynne; 1 juv. (BPBM), Mahunga Hiva Hiva, surface in front of Cave Q15-038, timed search at 1C (eastern-most collapse pit on southern extent near cave entrance), 20.VIII.2008, leg. J.J. Wynne; 3 ♂♂ (BPBM), Cave Q15-038, fern-moss garden (entrance zone), direct intuitive search, 4.XII.2008, leg. J.J. Wynne; 1 ♂, 2 ♀♀ (BPBM), Mahunga Hiva Hiva, Cave Q15-076/078, opportunistic collection, 4.VII.2009, leg. J.J. Wynne; 1 ♂, 2 ♀♀ (BPBM), Mahunga Hiva Hiva, Cave Q15-070, fern-moss garden (entrance zone), direct intuitive search, 13.VII.2009, leg. J.J. Wynne; 2 ♂♂, 1 ♀ (BPBM), Mahunga Hiva Hiva, Cave Q15-074, skylight entrance (1^st^ entrance NW of main entrance; entrance zone), opportunistic collection, 3.VII.2009, leg. J.J. Wynne; 1 juv. (BPBM), Mahunga Hiva Hiva, Cave Q15-067, fern-moss garden (entrance zone), direct intuitive search, 10.VII.2009, leg. J.J. Wynne; 1 ♀ (BPBM), Mahunga Hiva Hiva, Cave Q15-127, entrance zone, pitfall trap 1A, 5.VII.2009, leg. J.J. Wynne; 1 ♂ (BPBM), same data, pitfall traps 1B; 1 ♂, 1 ♀ (BPBM), Mahunga Hiva Hiva, surface grid 1, 27°06'53.1"S, 109°24'20.3"W, pitfall trap 3, 10.VII.2009, leg. J.J. Wynne; 1 ♂, 1 ♀ (BPBM), same location, pitfall trap 10, 10.VII.2009, leg. J.J. Wynne; 2 ♂♂, 5 ♀♀ (BPBM), same location, pitfall trap 12, 10.VII.2009, leg. J.J. Wynne.

###### Distribution.

Cosmopolitan species of western European origin.

##### Porcellio laevis

Taxon classificationAnimaliaIsopodaPorcellionidae

Latreille, 1804

###### Material examined.

Chile, Rapa Nui: 2 ♂♂ (AMNH 18362), VIII.1999, leg. C. Boyko, J. Tanacredi, S. Reanier, H. Tonnemacher and S. Lopez; 2 ♂♂, 1 juv. (AMNH 18363), Maunga Tangaroa, 20.VIII.1999, leg. C. Boyko, J. Tanacredi, S. Reanier, H. Tonnemacher and S. Lopez; 1 ♂, 1 ♀ (AMNH 18365), Cave Q15-074, location within cave not reported, 21.VIII.1999, leg. C. Boyko, J. Tanacredi, S. Reanier, H. Tonnemacher and S. Lopez; 2 ♂♂, 7 ♀♀ (AMNH 18361), La Pérouse Bay, 21.VIII.1999, leg. C. Boyko, J. Tanacredi, S. Reanier, H. Tonnemacher and S. Lopez.

###### Distribution.

Cosmopolitan species of Mediterranean origin.

### Family Armadillidiidae

#### Genus *Armadillidium* Brandt, 1831

##### Armadillidium vulgare

Taxon classificationAnimaliaIsopodaArmadillidiidae

(Latreille, 1804)

Armadillidium vulgare ; Fuentes, 1914: 315.

###### Material examined.

Chile, Rapa Nui: 1 ♂ (AMNH 18362), VIII.1999, leg. C. Boyko, J. Tanacredi, S. Reanier, H. Tonnemacher and S. Lopez; 6 ♂♂, 5 ♀♀ (AMNH 18365), Cave Q15-074, location within cave not reported, 21.VIII.1999, leg. C. Boyko, J. Tanacredi, S. Reanier, H. Tonnemacher and S. Lopez; 3 ♂♂, 9 ♀♀ (AMNH 18366), Hotel Hanga Roa, Hanga Roa, 21.VIII.1999, leg. C. Boyko, J. Tanacredi, S. Reanier, H. Tonnemacher and S. Lopez; 2 ♂♂, 2 ♀♀ (AMNH 18364), Poike region, 25.VIII.1999, leg. C. Boyko, J. Tanacredi, S. Reanier, H. Tonnemacher and S. Lopez; 1 ♂, 1 ♀ (BPBM), Mahunga Hiva Hiva, surface in front of Cave Q15-038, timed search at 1B (eastern-most collapse pit, southern extent near cave entrance), 20.VIII.2008, leg. J.J. Wynne; 1 ♀ (BPBM), Cave Q15-038, fern-moss garden (entrance zone), direct intuitive search, 21.XIII.2008, leg. J.J. Wynne; 2 ♀♀ (BPBM), Mahunga Hiva Hiva, surface grid 2, 27°06'41.3"S, 109°25'09.2"W, pitfall trap 23, 10.VII.2009, leg. J.J. Wynne; 1 ♂ (BPBM), surface grid 1, 27°06'53.1"S, 109°24'20.3"W, pitfall trap 17, 10.VII.2009, leg. J.J. Wynne; 3 ♀♀ (BPBM), same location, pitfall trap 19, 10.VII.2009, leg. J.J. Wynne; 1 ♂, 3 ♀♀ (BPBM), same location, pitfall trap 4, 10.VII.2009, leg. J.J. Wynne.

###### Distribution.

Cosmopolitan species of Mediterranean origin.

### Family Armadillidae

#### Genus *Venezillo* Verhoeff, 1928

##### Venezillo parvus

Taxon classificationAnimaliaIsopodaArmadillidae

(Budde-Lund, 1885)

###### Material examined.

Chile, Rapa Nui: 2 ♂♂ (BPBM), Mahunga Hiva Hiva, surface grid 2, 27°06'41.3"S, 109°25'09.2"W, pitfall trap 39, 10.VII.2009, leg. J.J. Wynne; 1 ♂ (BPBM), same location, pitfall trap 32, 10.VII.2009, leg. J.J. Wynne.

###### Distribution.

Widespread in tropical and subtropical regions. It has been introduced to European greenhouses. For diagnostic figures of this species see Schmidt (2003).

### Key to species of terrestrial isopods from Rapa Nui

**Table:** 

1	Antennal flagellum with >10 articles; eye with >100 ommatidia	***Ligia exotica***
–	Antennal flagellum with <6 articles, eye with <30 ommatidia	**2**
2	Antennal flagellum of 5 articles	***Styloniscus manuvaka***
–	Antennal flagellum of 3 or 2 articles	**3**
3	Antennal flagellum of 3 articles	***Hawaiioscia rapui***
–	Antennal flagellum of 2 articles	**4**
4	Body depigmented; eye consisting of a single ommatidium	***Trichorhina tomentosa***
–	Body pigmented; eye consisting of several ommatidia	**5**
5	Body slightly convex, unable to roll up into a ball	**6**
–	Body strongly convex, able to roll up into a perfect ball	**8**
6	Cephalon with a V-shaped suprantennal line; pereonite 1 with posterior margin straight and posterior corners rounded	***Porcellionides pruinosus***
–	Cephalon without suprantennal line; pereonite 1 with posterior margin more or less concave at sides and posterior corners right-angled or acute	**7**
7	Dorsal body surface smooth	***Porcellio laevis***
–	Dorsal body surface distinctly granulated	***Porcellio scaber***
8	Cephalon with a triangular frontal scutellum; telson trapezoidal; uropod exopodite flattened, filling the gap between telson and pleonite 5	***Armadillidium vulgare***
–	Cephalon with no frontal scutellum; telson hour-glass shaped; uropod protopodite flattened, filling the gap between telson and pleonite 5	***Venezillo parvus***

## Discussion

Nine species of terrestrial isopods are known from Rapa Nui: *Ligia exotica*, *Styloniscus manuvaka* sp. n., *Hawaiioscia rapui* sp. n., *Trichorhina tomentosa*, *Porcellionides pruinosus*, *Porcellio laevis*, *Porcellio scaber*, *Armadillidium vulgare*, and *Venezillo parvus*.

Only one species (*Ligia exotica*) is littoral, halophilic, and widely distributed along coastal habitats in the tropics. We have not examined any specimens belonging to this species and its identification needs to be confirmed. Littoral habitats have not been adequately sampled on Rapa Nui and other littoral species may also be present on the island. Two species (*Trichorhina tomentosa* and *Venezillo parvus*) have a wide distribution in the tropics, and four species of European or Mediterranean origin (*Porcellionides pruinosus*, *Porcellio laevis*, *Porcellio scaber*, and *Armadillidium vulgare*) are now cosmopolitan. All of these species were introduced to Rapa Nui due to human activities. *Styloniscus manuvaka* sp. n. and *Hawaiioscia rapui* sp. n. are Polynesian and Rapa Nui endemics, respectively.

Given that few native arthropod species remain on Rapa Nui (Wynne et al. 2014), the two new isopod species are a significant contribution to the island’s natural history. Together with the other eight endemics described by Bernard et al. (2015), Mockford and Wynne (2013) and Jordana and Baquero (2008), these disturbance relicts have persisted despite several hundred years of extreme environmental change and interactions with non-native species (Wynne et al. 2014).

Despite their persistence, these endemic species are considered imperiled (Wynne et al. 2014). *Styloniscus manuvaka* and *Hawaiioscia rapui* may be operating under extinction debts (Triantis et al. 2010). This may occur once a population has become isolated following a significant environmental perturbation, such as habitat loss or fragmentation (Tilman et al. 1994). Habitat loss has occurred dramatically and at an island-wide scale on Rapa Nui. Both *Styloniscus manuvaka* and *Hawaiioscia rapui* were detected in low numbers. Neither of these species were detected during earlier inventory work (see Fuentes 1914, Olalquiaga 1946, Kuschel 1963, Campos and Peña 1973) or our surface sampling effort.

Further, the combined effects of global climate change and interactions with non-native species may further threaten the persistence of these endemic isopods. Competition with non-native species has been identified as threatening the persistence of surface-dwelling endemic arthropods on other island ecosystems (see Chown et al. 2007, Fordham and Brook 2010, Vitousek et al. 1997). Increased drought conditions are predicted for the sub-tropics (IPCC 2013) and other Polynesian islands (Chu et al. 2010). We also know non-native species represent the majority of known arthropods on Rapa Nui. For example, of the seven known non-native isopod species, the cosmopolitan *Porcellio scaber* was detected in the greatest numbers in both surface sampling and within caves (Wynne et al. 2014). Additionally, *Porcellio scaber* is a well-established non-native species being first detected by Fuentes (1914). In Hawai‘i, *Porcellio scaber* is considered to be an invasive species and one of the most damaging non-native arthropods in the native ecosystems (Howarth et al. 2001).

Conservation and management of these endemic terrestrial isopods (as well as the other endemic species) and their habitats should be a high priority for the Rapanui community, policy makers and resource managers. Given the concerns associated with global climate change and non-native invasive species, a captive breeding program of these new species is recommended. Captive breeding of isopods is relatively easy and inexpensive (Sutton 1972). Such a program may be developed in collaboration with CONAF, Museo Antropológico P. Sebastián Englert de Rapa Nui and potentially secondary school classrooms on the island. By captively breeding these animals in a variety of locations, their long-term persistence may be somewhat safeguarded, and will facilitate the establishment of viable populations for future reintroduction efforts. Additionally, this will provide an opportunity for researchers to obtain information associated with the life history characteristics of these endemic species. Also, once large captive populations are established, experiments examining competition with the non-native isopods may be performed.

Northup and Welbourn (1997) proposed that moss garden habitats in New Mexico lava tube caves may serve as a source habitat for arthropods colonizing cave deep zones. Fern-moss gardens within Rapa Nui caves may provide this same function. All known congeners of *Hawaiioscia rapui* sp. n. are cave-adapted isopods from the Hawaiian Islands. *Hawaiioscia rapui* sp. n. was detected within both entrance and twilight zones. If this species persists, it is possible parapatric speciation may occur as has been suggested for other *Hawaiioscia* species from the Hawaiian Islands (Rivera et al. 2002).

Finally, we know little concerning the distributions of these endemic isopods. We recommend additional surveys be conducted in other caves on the island, as well as in other habitats likely to support terrestrial isopods (and endemic arthropods, in general). This final step will provide resource managers with the ability to better characterize endemic isopod habitat, and to further improve our understanding of the distribution of these animals on Rapa Nui.

## Supplementary Material

XML Treatment for Ligia exotica

XML Treatment for Styloniscus manuvaka

XML Treatment for Hawaiioscia rapui

XML Treatment for Trichorhina tomentosa

XML Treatment for Porcellionides pruinosus

XML Treatment for Porcellio scaber

XML Treatment for Porcellio laevis

XML Treatment for Armadillidium vulgare

XML Treatment for Venezillo parvus
